# The Effect of Osmolytes on Protein Fibrillation

**DOI:** 10.3390/ijms13033801

**Published:** 2012-03-21

**Authors:** Francesca Macchi, Maike Eisenkolb, Hans Kiefer, Daniel E. Otzen

**Affiliations:** 1 iNANO, Center for Insoluble Protein Structures (inSPIN), Department of Molecular Biology and Genetics, Aarhus University, Gustav Wieds Vej 10C, DK-8000 Aarhus C, Denmark; E-Mail: francesca@inano.au.dk; 2 Hochschule Biberach, Pharmaceutical Biotechnology, Hubertus-Liebrecht-Str. 35, D-88400 Biberach, Germany; E-Mails: eisenkolb@hochschule-bc.de; kiefer@hochschule-bc.de

**Keywords:** glucagon, amyloid, taurine, polymorphism, fibrillation mechanism

## Abstract

Osmolytes are small molecules that are exploited by cells as a protective system against stress conditions. They favour compact protein states which makes them stabilize globular proteins *in vitro* and promote folding. Conversely, this preference for compact states promotes aggregation of unstructured proteins. Here we combine a brief review of the effect of osmolytes on protein fibrillation with a report of the effect of osmolytes on the unstructured peptide hormone glucagon. Our results show that osmolytes either accelerate the fibrillation kinetics or leave them unaffected, with the exception of the osmolyte taurine. Furthermore, the osmolytes that affected the shape of the fibrillation time profile led to fibrils with different structure as revealed by CD. The structural changes induced by Pro, Ser and choline-*O*-sulfate could be due to specific osmolytes binding to the peptides, stabilizing an otherwise labile fibrillation intermediate.

## 1. Introduction

### 1.1. Osmolytes: Chemical Chaperones Which Can Stabilize or Destabilize Proteins

Osmolytes are small organic molecules that have evolved throughout various taxa and increase the ability of cells to react to osmotic stress. In contrast to inorganic ions, which can also be used for adaptation to extracellular stress, osmolytes do not negatively interfere with structure or function of biological macromolecules. For example, inorganic ions have been shown to reduce enzymatic activity in the case of phosphoenolpyruvate (PEP) for pyruvatekinase (PK) of the crab, while osmolytes do not interfere with its activity [1]. Similarly, the disruption of the native myofilament architecture induced by KC1 and NaCI can be prevented by addition of the osmolyte trimethyl amine oxide (TMAO) [2].

Most osmolyte compounds can be divided into three chemical classes: polyhydric alcohols and sugars (polyols), amino acids and their derivatives and methyl ammonium compounds [3]. Osmolytes are widely used to stabilize and facilitate protein folding since they can act as “chemical chaperones” [4,5]. For many proteins, the melting temperature *T*_m_ increases with the addition of osmolytes in a concentration-dependent manner [3,5]. Osmolytes are generally thought to act by preferential surface exclusion. This means that they destabilize expanded states such as the unfolded state to a greater extent than more compact states (e.g., the native state) [6,7]. Thus in practice osmolytes stabilize globular proteins by favouring compaction. However, this phenomenon can be a disadvantage in the case of intrinsically disordered proteins where compaction can promote intermolecular aggregation.

Note however that osmolytes, depending on solvent conditions can also decrease protein melting temperatures [8]. This is not too surprising, as evolutionary selection will favour the production of osmolytes only if they lead to enhanced protein function at ambient temperature. There is no evolutionary pressure for increased stability at physiologically irrelevant elevated temperatures. As a result, some osmolytes thermodynamically destabilize folded proteins but reduce aggregation at the same time, the most prominent example being urea [9,10]. This osmolyte, widely used for its destabilizing effects for protein stability and folding studies *in vitro*, is used by many organisms in combination with methylamines (ratio 2:1) for an optimal functionality of its enzymes [11]. It is therefore important to distinguish between “stabilization effects” that act on *T*_m_ and those that act on aggregation kinetics. Similarly, an increase in *T*_m_ does not necessarily relate to the reversibility of the folding process. For example, the *T*_m_ of lysozyme unfolding can be increased by 22 °C by addition of sarcosine, but as complete unfolding takes place even in the presence of osmolyte, the protein aggregates irreversibly, while other proteins retain their reversible unfolding [12].

Note also that most studies on osmolytes investigate their effect on thermodynamic stability [7,13]. This does not necessarily correlate with “stabilization” in the sense of preserving functionality over extended periods. The latter can result partially or completely from kinetic inhibition of aggregation and may have a large impact in industrial applications. Data about osmolytes affecting kinetic stability are scarce: Nayak *et al.* [14] determined fibrillation and nucleation rate constants of insulin in the presence of polyols. Osmolyte influence on RNAse A enzymatic function has been reported by Jamal *et al.* [15]. However, the molecular processes leading to the changes in *K*_m_ and *k*_cat_ were not established.

One parameter strongly influencing osmolyte impact is pH. According to Singh *et al.* [8], polyols show a more potent stabilizing effect towards proteins’ native state at low pH. A possible explanation would be that at low pH, carboxyl groups in Glu and Asp become protonated, making hydrogen bonds with polyols less favorable than at high pH, where carboxyl groups are deprotonated and negatively charged. As a result preferential surface exclusion of polyols and consequent protein stabilization increases at low pH [16]. The stabilizing effect of methylamines on the other hand has been found to be most pronounced at neutral pH, while they can be destabilizing at low pH. [15]. This observation has been attributed to the fact that at neutral pH, zwitterionic methylamines are excluded from the protein surface, *i.e.*, act as stabilizers, while in their positively charged form at low pH, they will directly interact with the protein backbone, preferentially stabilizing the denatured state and decreasing the free energy of unfolding Δ*G*_D-N_. This effect has been described for TMAO and Betaine [17–19]. A similar effect would be expected for amino acids and their derivatives. However, those compounds were found to stabilize the native state proteins almost independent of pH. It appears that here, effects of pH on Δ*G*_D-N_ resulting from direct protein-osmolyte interaction and surface exclusion are balanced [15]. Protein destabilization may also occur at very high osmolyte concentrations [12,20], where the modestly favorable interactions between apolar parts of the osmolyte and hydrophobic side chains become more significant, resulting in exposure of hydrophobic residues and unfolding [12]. The mechanistic bases for other properties of osmolytes are still subject to debate: For instance, it was reported that higher molecular weight polyols stabilize proteins better than their low MW counterparts [21]. There are also clear indications that part of the action of osmolytes can be protein-specific: e.g., glycine betaine at physiological pH strongly stabilizes RNase A but leads to aggregation or partial unfolding of green fluorescent protein [18,19].

### 1.2. Osmolytes and Protein Fibrillation

Despite the large number of studies investigating the effect of osmolytes on protein folding, few investigations have been performed on the effect of osmolytes on protein fibrillation. Here we provide a brief review of the published work on the topic. We emphasize that the paucity of systematic investigations makes it difficult to make generalizations about the impact of different osmolyte classes on the fibrillation process, and we hope that the present work (see below) may inspire further work to mitigate this state of affairs.

In general it is difficult to predict how osmolytes affect protein aggregation. Some osmolytes such as amino acids and methylamines, may bind relatively strongly to proteins or can alter protein hydration patterns and may thus influence protein-protein interactions and the structure of the ensuing aggregate, which can vary from oligomer(s) to fibrils to amorphous aggregates. TMAO for example, induces Aβ oligomerization but encourages tau fibrillation [22] though both proteins are natively unfolded.

For globular proteins, destabilization of the most expanded state leads to correct folding, while in the case of intrinsically disordered proteins (IDPs) the same process can promote aggregation. This can occur in two situations. Aggregation of the IDP sometimes requires formation of a compact monomeric state that contains elements of structure favoring intermolecular contacts (cfr. the stimulation of aggregation of the IDP α-synuclein by salts and low pH [23] and similar observations for other proteins [22,24]). Alternatively, the oligomeric or fibrillar state is simply favored because it is more compact than the exposed denatured state. An example of such an effect is given by the amino acid proline. This osmolyte inhibits aggregation of globular proteins such as lysozyme [25] and P39A cellular retinoic acid-binding protein [26], while mutant huntingtin exon 1 aggregates into amorphous aggregates [27] and we report that Pro promotes fibrillation of glucagon (see below).

The intrinsically disordered protein α-synuclein, involved in Parkinson’s Disease, forms a relatively heterogeneous ensemble of compact oligomeric structures in the presence of 3 M TMAO, while at lower TMAO concentrations the formation of a partially folded intermediate accelerates fibrillation [28]. Similarly, 1 M glucose induces collapse of α-synuclein [29] (Table 1). TMAO also accelerates the fibrillation of *S*-carboxymethylated α-lactalbumin [30] and induces oligomerization of the prion protein [31] but inhibits conversion to the amyloidogenic form of the prion protein *in vivo* [32]. Oligomerization by TMAO is also observed for the amyloid β peptide Aβ involved in Alzheimer’s Disease [33], and TMAO increases the secondary structure content of the flexible and prion-determining region of Sup35p [34].

The fact that osmolytes can inhibit fibrillation but induce oligomerization should be taken into account if the ultimate purpose is a reduction or complete inhibition of the aggregates’ toxicity: in all cases, the properties of the ensuing aggregates have to be studied in detail. A good example is provided by Aβ. Galactose and mannose induce the aggregation of Aβ40 and Aβ42 into mature fibrils, while glucose, sucrose and fructose make them oligomerize [24]. Other osmolytes, such as glycine and taurine, accelerate Aβ40 fibril formation [35] while trehalose completely inhibits its aggregation. As for Aβ42, 50 mM trehalose makes the peptide form toxic oligomers [36] while 250 mM trehalose makes it fibrillate slower than in the absence of chemical chaperones [37]. Other studies instead reported lower toxicity and reduced protein aggregation in the presence of trehalose *in vivo* [38,39].

Several osmolytes have been reported to inhibit fibrillation, while favouring the formation of oligomers or amorphous aggregates in the case of intrinsically disordered proteins (Table 1). This could be due to the general mechanism of destabilization of the unfolded state caused by the preferential exclusion from the protein surface, together with possible protein-specific interactions. In this context the amorphous aggregates induced by the osmolytes represent an alternative way to render the protein compact, which may prevail over fibrillar aggregates for reasons of kinetic accessibility.

A cautionary note is required regarding the use of ThT fluorescence intensities to quantify the amount of formed fibrils, as presented in [36,40]. In fact, ThT fluorescence intensity could potentially be affected by interactions between ThT and the osmolyte, which is always present at many thousand-fold higher concentrations than ThT, as well as by competitive osmolyte binding on the fibril surface [41,42]. Furthermore, osmolytes can induce fibrillar polymorphism, as we report for glucagon (see below). In this case, ThT intensities are influenced by the fibrils’ morphology [43] and therefore cannot be used to quantify the amount of peptide in the fibrillar form. For these reasons, control experiments to evaluate possible fluorescence quenching effects and parallel use of complementary techniques to evaluate the amount of fibrillated material (e.g., centrifugation to determine the amount of aggregated protein and CD/FTIR to ascertain possible changes in structure) are always recommended.

### 1.3. Glucagon: A Model System for Fibrillation

As a complete and systematic study of the effects of osmolytes on intrinsically disordered proteins is lacking, we performed a study on the natively unfolded peptide glucagon in which we compare the effects of many osmolytes belonging to the three different classes. Glucagon is an intrinsically disordered peptide hormone, which has been shown to readily fibrillate *in vitro* into fibrillar aggregates with different structure and morphology depending on the conditions used for fibrillation, such as protein concentration and salt [49]. Under acidic conditions, glucagon fibrillation is particularly sensitive to the presence of anions, with effects following the electroselectivity series, rather than the Hofmeister series. In particular, addition of sulfates results in faster fibrillation into a stable fibrillar structure showing an “α-like” CD spectrum. Our results confirm that proteins lacking a globular structure can aggregate more easily in the presence of osmolytes and that these cosolutes can affect glucagon fibrils’ structure.

## 2. Results

### 2.1. Glucagon Fibrillation Is Either Accelerated or Unaffected by Osmolytes

Here we describe a systematic study of the effects of osmolytes on glucagon fibrillation. A representative sample of osmolytes was selected from Table (6.1) in Hochachka *et al.* [50]. For the polyols class we present results for trehalose, sucrose, mannitol, sorbitol, erythritol, glucose, myo-inositol and glycerol; for the class containing the amino acids and their derivatives we present results for Pro, Ala, Gly, Ser, taurine, ectoine and OH-ectoine; for the methylamines class we report data for glycine betaine, sarcosine and choline-*O*-sulfate (COS).

**Figure 1:**
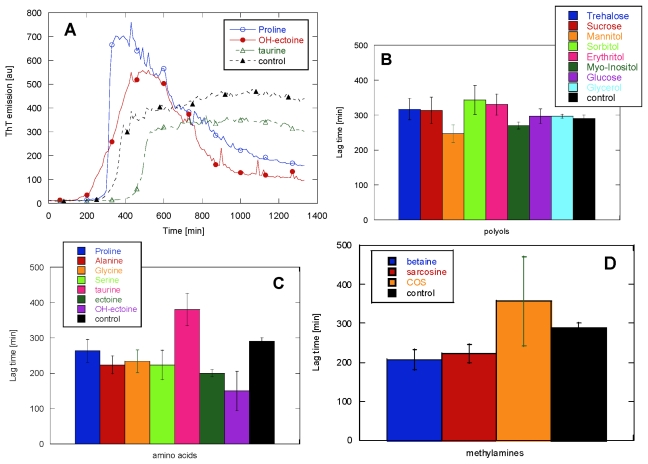
(**A**) Typical fibrillation kinetic trace in buffer alone and in the presence of the osmolytes taurine, OH-ectoine and proline. (**B**) Lag times for glucagon fibrillation in the presence of 125 mM polyols, which leaves glucagon fibrillation mostly unaffected. (**C**) 125 mM of the amino acids and ectoines accelerate glucagon fibrillation, while taurine is the only osmolyte that shows a longer lag time compared to the control. (**D**) 125 mM methylamines generally shorten the lag time for glucagon fibrillation.

To explore the kinetics of glucagon fibrillation in the presence of osmolytes, ThT emission was measured over time for samples with a constant glucagon concentration (1 mg/mL) and 125–400 mM osmolytes. The raw ThT data immediately show that no osmolyte was able to inhibit glucagon fibrillation. Figure 1 shows the effect of including 125 mM osmolyte in the glucagon solution on the fibrillation lag time. The sugars and polyols class generally show very little effect on the lag time of fibrillation. The amino acids class generally reduce the lag time, particularly Ala, Gly and Ser. Pro has a very modest effect, while ectoines (ectoine and OH-ectoine) significantly decrease the lag time. The only osmolyte that showed a protective effect by increasing the lag time is taurine. In the methylamines class, betaine and sarcosine decrease the lag time.

**Figure 2:**
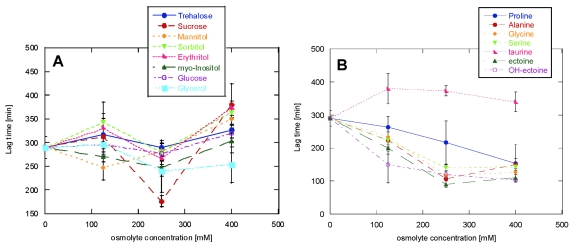

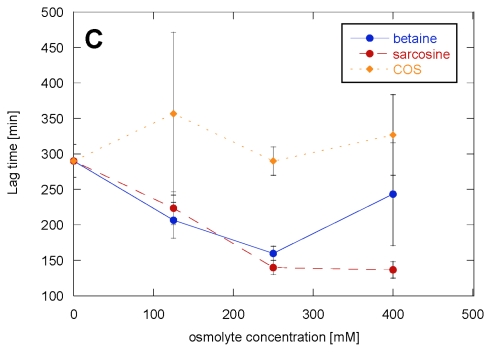
Effect of osmolyte concentration on the lag time for glucagon fibrillation, on the different cosolute classes: (**A**) polyols, (**B**) amino acids and their derivatives, (**C**) methylamines.

Increasing concentrations of osmolytes generally only had a modest effect on the onset of fibrillation (Figure 2). The polyols class, which at 125 mM osmolyte showed very little effect, did not show any change when the cosolute concentration was doubled or increased up to 400 mM. Increasing the amino acids concentration produced only a very modest, and often statistically insignificant, decrease in the lag time. Similar behaviour was shown for the methylamines class: the only osmolyte that gave a significant reduction of the lag time upon increasing its concentration from 125 to 250 mM was sarcosine.

**Figure 3:**
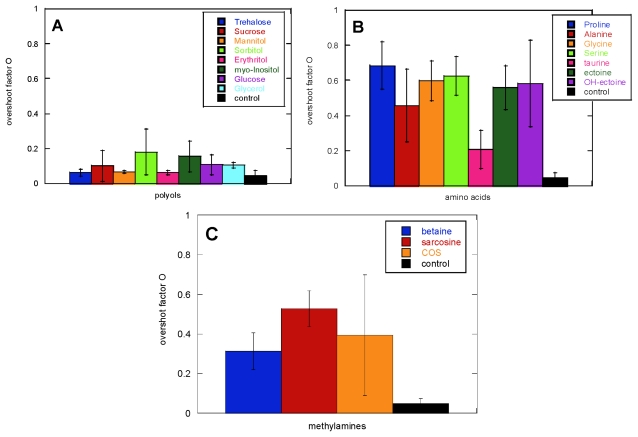
Effect of 125 mM osmolytes on the appearance of the ThT kinetic traces, as shown by the *O* parameter.

The shape of the ThT time profile showed more variation among the osmolytes than the lag time (Figure 1A). Osmolytes with limited ability to change the lag time gave rise to very different fibrillation kinetics, as shown by the overshoot parameter *O* described in Equation 1 (Figure 3). For example, proline showed only a 10% reduction in the lag time, while it was able to increase the *O* factor from 0.1 to 0.7.

The overshoot phenomenon in glucagon fibrillation was first reported by Pedersen *et al.* [51] and confirmed by more recent studies [52]. This overshoot correlated with the transient accumulation of a high ThT-staining fibrillation intermediate (type B fibrils), which subsequently interconverts into the most stable fibrillar form (low stress or type B fibrils). The shape of the ThT signal can therefore be used as an indication of the pathway taken by the fibrillation reaction. A lack of overshoot (seen in the absence of osmolytes) is consistent with a lack of type B fibrils; rather, glucagon fibrillates directly to the so-called high-stress fibrils [53]. This is a mechanism that was specifically investigated in the case of glucagon and might not be transferable to other proteins without prior tests.

**Figure 4:**
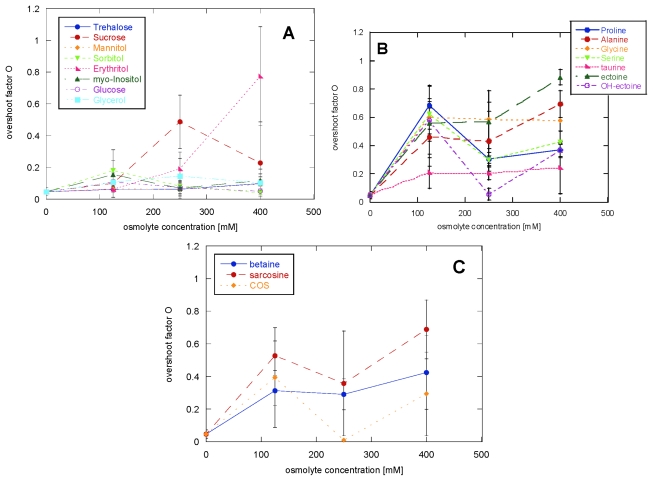
Effect of increasing osmolyte concentrations on the *O* parameter for (**A**) polyols, (**B**) amino acids and (**C**) methylamines.

Adding 125 mM amino acids led to an increase of the *O* factor, with the exception of taurine. The more prominent presence of an overshoot was also seen when adding betaine and sarcosine, while the polyols left the *O* factor mostly unaffected. Increasing the polyol concentration led in the case of erythritol to an increase in the *O* factor and a decrease in reproducibility; the other polyols again showed no effects even at 400 mM (Figure 4A). Increasing concentrations of Gly, Ala, taurine and OH-ectoine gave no additional effect, while Pro and Ser caused a slight decrease in the *O* factor and ectoine a modest increase (Figure 4B). Increasing the concentration of methylamines up to 400 mM gave no further change in the *O* factor (Figure 4C).

### 2.2. CD Reveals Glucagon Polymorphism in the Presence of Osmolytes

To evaluate whether the differences in lag time and overshoot factor indeed could be attributed to the formation of different fibrillar types, we acquired CD spectra of the samples in the presence of 125 mM osmolytes (except for ectoine and OH ectoine where high background absorbance interfered with spectral recording).

**Figure 5:**
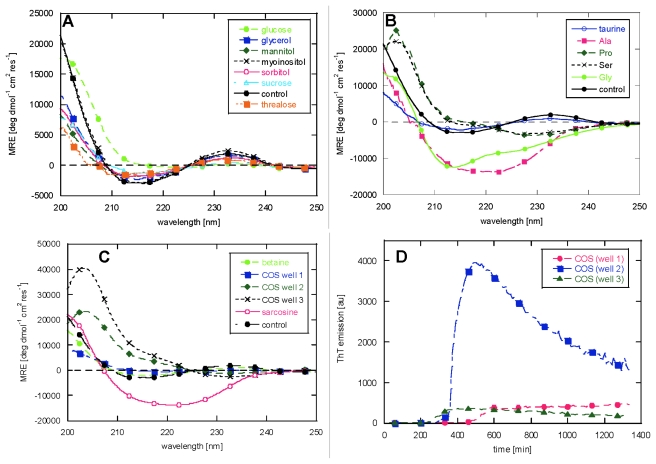
CD spectra of glucagon samples in the presence of 125 mM (**A**) polyols, (**B**) amino acids and (**C**) methylamines. It was not possible to acquire CD spectra of the ectoine samples, as their absorbance saturated the detector, while strong dilution would not allow the fibril detection. The three replica wells of the choline-*O*-sulfate (COS) samples are displayed as they showed different spectra which correlated with their fibrillation traces (panel **D**).

All our fibrillation studies are carried out under agitated conditions (shaking at 720 rpm). Accordingly, the osmolyte-free fibrils show the β-sheet like spectrum observed for glucagon fibrils in the presence of high mechanical stress [54] with a negative peak at 215 nm and a positive peak at 232 nm, that we attribute to aromatic contributions to the CD spectrum [55]. Adding polyols not only leaves the ThT traces unaffected, as shown both for the lag time and the *O* parameter, but also shows almost no effect on the CD spectra, except for a small red-shift of the minimum and reduction of the intensity of minimum and maximum in the presence of glucose (Figure 5A).

Adding amino acids or their derivatives dramatically changes the fibrils’ morphology (Figure 5B). Despite the high degree of shaking, Pro and Ser lead to CD spectra typical of glucagon fibrils in the presence of low mechanical stress [54], also known as type B fibrils [43], with a positive peak at 203 nm and a negative peak at 230 nm. Remarkably, Ala shows a spectrum typical of type D fibrils [43] grown in the presence of high NaCl concentration, characterized by a broad minimum at around 220 nm. Gly was previously used at 50 mM (pH 2.5) as standard buffer in previous studies [43,54,56], but 175 mM Gly made glucagon fibrillate into a β-sheet-like structure, with a shoulder at around 225 nm; taurine instead shows the same CD spectrum as the control (negative peak at 215 nm and a positive peak at 232 nm).

The methylamines created variations in the spectra as well: while betaine shows a spectrum identical to osmolyte-free glucagon, sarcosine shows a low stress type B spectrum. An anomalous behaviour was shown only in the case of Choline-*O*-Sulfate (COS): the three replica wells showed different spectra (Figure 5C) which tie in with the variation in the kinetic time profiles (Figure 5D). Two of the three wells gave the low stress fibrillar type, though with different peak intensities, while the third well shows a flat spectrum with a very shallow minimum at 218 nm, which remains at MRE values just below zero up to 250 nm.

The effects of the osmolytes, both in terms of kinetics and structure are summarized in Table 1 (column “Glucagon”).

## 3. Discussion

In this study we have examined the effect of osmolytes on glucagon fibrillation. Glucagon is a short peptide lacking a folded native structure; for the peptide to aggregate, we have previously proposed that a partially folded intermediate must form in which residues 6–10 and 23–27 come in close vicinity to each other [57]. Thus fibrillation requires the peptide to become more compact. We observe that different types of osmolytes cannot inhibit aggregation, similarly to results reported for other intrinsically disordered proteins, such as Aβ and tau [22,24]. With few exceptions, osmolytes promote faster fibrillation of glucagon or have no effect at all. Exceptions also occur for other peptides and proteins: trehalose was able to inhibit Aβ fibrillation [36] while polyols slowed down the fibrillation of insulin [14]. The only osmolyte that showed a delay of glucagon’s fibrillation onset and no effect on glucagon fibrillar morphology is the amino acid derivative taurine, which was previously reported to accelerate Aβ aggregation [35] and to generally promote protein compaction [15]. Other amino acids derivatives, such as ectoine, had the opposite effect to taurine. Thus the same osmolyte can have different effects depending on the specific protein used in the study.

Each protein can therefore have a reduced or increased tendency to fibrillate, depending on the osmolyte used. Some osmolytes indeed have similar effects on very different proteins. The polyol fructose has no effect on the aggregation behaviour of folded proteins as insulin [14] or the unstructured peptide Aβ [24], while osmolytes such as trehalose and TMAO mostly show inhibitory effects on the formation of amyloid-like fibrils formed by different proteins (Table 1).

Osmolytes belonging to the polyol class showed very little or no effect on glucagon fibrillation kinetics and CD spectra, even though they are reported to be most effective at low pH [8], as used in this study. Amino acids and methylamines on the other hand showed an effect both on the fibrillation kinetics and on the fibrils’ structure. Moreover, methylamines are reported to destabilize at low pH [15], and in this study they lead to faster aggregation of glucagon. Amino acids are thought to balance hydration (preferential exclusion) and binding effects. In particular, the polyols class is known to act through these hydration effects. However, these effects clearly have little influence on the fibrillation of glucagon. This leaves osmolyte binding as a possible mediator of glucagon fibril polymorphism.

The overshoot factor was a more sensitive detector than the lag time for the presence of different fibrillar morphologies for the different samples analyzed. For example, while Pro showed a 10% reduction of the lag time, changing completely the CD spectrum (*O* parameter going from 0.1 in the absence of osmolyte to 0.7 in the presence of Pro), mannitol showed a 15% reduction of the lag time, but left the *O* parameter and the CD spectrum unaffected. Generally, an *O* parameter <0.4 leads to an osmolyte-free CD spectrum while *O* parameters >0.4 changes the morphology towards other fibrillar types, such as the low stress (also known as type B) and type D fibrils [43]. In the case of COS there is a particularly close correlation between the shape of the curve and the resulting morphology. This osmolyte gives the lowest reproducibility, both in terms of lag time and overshoot factor, and different kinetic profiles (Figure 5D) lead to different fibril morphologies according to CD spectra (Figure 5C).

**Figure 6:**
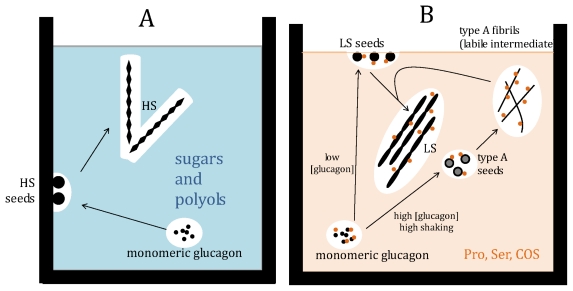
Suggested fibrillation mechanism. Left panel: the absence of osmolytes leads to high stress (HS) fibrils. Right panel: the presence of Pro, Ser or COS is suggested to lead to the formation of the labile type A intermediate as the osmolytes bind glucagon, “shielding” it from the high stress conditions. The resulting fibrils are here called low stress (LS).

The role of the osmolytes on the selection of the fibril structure is a fascinating issue. Pro, Ser and COS modify the structure of glucagon fibrils from the typical structure in the presence of high mechanical stress to the one present in the exact same conditions, but under low stress. This can be rationalized by a protection effect exerted by the osmolytes on glucagon, “shielding” the effect of mechanical perturbation, particularly as amino acids preferentially bind to proteins [8] (Figure 6). This would happen as osmolytes could allow the formation of the labile intermediate, which is required for the formation of low stress fibrils, even under high stress conditions, in which the formation of the labile intermediate is normally inhibited and therefore leads to another type of fibril [54].

It is difficult to explain the appearance of the type D fibrils in the presence of Ala and sarcosine, but we suggest that specific interactions between the peptide and these chemical chaperones could intervene in the fibrillation process, thus leading to another structure.

The very low concentration dependence on the effect of osmolytes generally makes their effect resemble electrostatic interactions where the effect saturates at an ionic strength corresponding to 100–200 mM, rather than a crowding effect, for which increasing osmolyte concentration will have a increasing effect at concentrations up to several molar [58,59].

In summary, it is evident that neither osmolytes in general nor those belonging to a particular compound class influence glucagon aggregation in a uniform manner. The distinct effects of compounds such as taurine, COS or Ala more likely result from direct interaction of the compounds with the protein than from non-specific solvent effects. Direct interaction might also be the reason for effects observed here that are not consistent with other published work, simply because they could be protein-specific. Since the molecular basis of aggregation is diverse, more model systems able to distinguish between those pathways will be needed to obtain a clearer picture. Also, larger sets of osmolytes as well as model proteins need to be screened to draw conclusions on their general mechanism of action, due to the specificity of some osmolyte-protein interactions.

## 4. Experimental Section

### 4.1. Glucagon Fibrillation in the Plate Reader

Glucagon powder was dissolved in 10 mM HCl (pH 2) and filtered through a 0.2 μm filter. The actual glucagon concentration in solution was determined from absorbance measurements on a Nanodrop (Thermo Fisher Scientific, Waltham, MA) using an extinction coefficient ɛ_280 nm_^1mg/mL^ = 2.369. The stock solution was subsequently diluted in 50 mM Gly buffer pH 2.5 containing 40 μM ThT, with or without the presence of osmolytes, to a final glucagon concentration of 1 mg/mL.

Solutions were transferred to a 96 wells plate with transparent bottoms for fluorescence reading (Nunc, Thermo Scientific, Roskilde, Denmark), sealed with transparent tape (Nunc) to avoid evaporation. Each condition was present in three replica wells.

The plates were incubated in a Tecan GeniosPro plate reader at 29 °C. ThT emission was measured (excitation wavelength 448 nm, emission 485 nm) every 10 min. During each interval linear shaking was performed at 720 rpm for 8 min.

To analyze the kinetic profiles, we extracted the lag time for all samples. The lag time was defined as the time required for the development of 5% of the maximum fluorescence intensity. We also calculated an overshoot factor O using the following equation:

(1) $$O=\frac{M-E}{M-i}$$

in which *M* is the maximum ThT fluorescence, *i* is the initial ThT fluorescence and *E* is the end point fluorescence value, in order to describe the shape of the kinetic trace, where *O* = 0 indicates the complete absence of overshoot and *O* = 1 an overshoot that leads back to the initial intensity value. Errors were calculated as standard deviations.

### 4.2. Circular Dichroism

Fibrillated glucagon samples with or without osmolyte presence were sonicated for 2 s with a Bandelin Sonopuls sonicator (Bandelin Electronic GmbH, Germany) to disperse aggregates efficiently, and subsequently diluted to a final concentration of 0.2 mg/mL glucagon in 25 mM glycine buffer. The samples were measured on a Jasco J-810 (Tokyo, Japan) spectrometer using a 1 mm quartz cuvette (Hellma GmbH, Germany). At least 5 accumulations were acquired, using a scanning speed of 100 nm/min and 0.2 nm data pitch. The data are reported as Mean Residue Ellipticity: MRE = 100θ/(*cdN*), where *θ* is the measured ellipticity in degrees, *c* is the protein concentration in mol/L, *d* is the light path in cm, and *N* is the number of residues. Background spectra including appropriate concentrations of osmolytes are subtracted from each spectrum.

The CD spectrum shown for the control is the one reported for glucagon in the presence of high mechanical stress [54]. Unlike the previous study [54], no glass bead was added. We attribute the formation of high stress fibrils to the usage of a new protein batch compared to the two batches used previously [54]. To facilitate comparison, all experiments were performed with the same batch.

## 5. Conclutions

It is evident that neither osmolytes in general nor those belonging to a particular compound class influence glucagon aggregation in a uniform manner. The distinct effects of compounds such as taurine, COS or Ala more likely result from direct interaction of the compounds with the protein than from non-specific solvent effects. Direct interaction might also be the reason for effects observed here that are not consistent with other published work, simply because they could be protein-specific. Since the molecular basis of aggregation is diverse, more model systems able to distinguish between those pathways will be needed to obtain a clearer picture. Also, larger sets of osmolytes as well as model proteins need to be screened to draw conclusions on their general mechanism of action, due to the specificity of some osmolyte-protein interactions.

## Figures and Tables

**Table 1:** Effect of osmolytes addition to several fibrillating proteins.

Osmolyte	Aβ40	Aβ42	α-synuclein	polyQ	Immunoglobulin light chains	Glucagon 1	insulin	lysozyme
**Glucose**	oligomerization [24]	oligomerization [24]	collapse			no effect	minor effect [14]	
**Sucrose**	oligomerization [24]	oligomerization [24]				no effect	no/minor effect [14,44]	reduced aggregation [25,45]
**Fructose**	no effect[24]	no effect [24]					minor effect [14]	
**Galactose**	induced fibrillation [24]	induced fibrillation [24]						
**Mannose**	induced fibrillation [24]	induced fibrillation [24]						
**Sorbitol**					longer lag, more stable fibrils [46]	no effect		
**Glycerol**	oligomerization [33]					no effect		no effect [25]
**Trehalose**	slower/no fibrillation [36,37]	oligomerization (toxic) [36]		reduced aggregation in mice [47]		no effect	reduced/slower fibrillation [14,44]	increasing B^1^ [48], reduced aggregation [45]
**Gly**	faster fibrillation [35]					faster fibrilation		no effect [25]
**Pro**				amorphous aggregates [27]		polymorphism		inhibits aggregation [25]
**Taurine**	faster fibrillation [35]					slower fibrillation		
**Ectoine**		longer lag time, more oligomers, lower toxicity[40]				faster fibrilation	highly reduced fibrillation [44]	
**Betaine**				faster fibrillation [27]	longer lag, more stable fibrils [46]	faster fibrilation	highly reduced fibrillation [44]	increasing B^2^ [48]
**Sarcosine**						faster fibrillation, polymorphism		Tm of unfolding increases by 22°, but the unfolded lysozyme aggregates [12]
**TMAO**	little effect [37]; oligomerization [33]		folded oligomer/enhanced fibrillation [28]	amorphous aggregates [27]				

Notes:

1 Based on results in this study.

2 B: osmotic second virial coefficient.

An increasing value means more attractive forces between molecules.

## References

[1] Bowlus R.D., Somero G.N. (1979). Solute compatibility with enzyme function and structure: Rationales for the selection of osmotic agents and end-products of anaerobic metabolism in marine invertebrates. J. Exp. Zool.

[2] Clark M.E., Hinke J.A.M., Todd M.E. (1981). Studies on water in barnacle muscle fibres II. Role of ions and organic solutes in swelling of chemically-skinned fibres. J. Exp. Biol.

[3] Yancey P.H., Clark M.E., Hand S.C., Bowlus R.D., Somero G.N. (1982). Living with water stress: Evolution of osmolyte systems. Science.

[4] Welch W.J., Brown C.R. (1996). Influence of molecular and chemical chaperones on protein folding. Cell Stress Chaperones.

[5] Meng F.-G., Park Y.-D., Zhou H.-M. (2001). Role of proline, glycerol, and heparin as protein folding aids during refolding of rabbit muscle creatine kinase. Int. J. Biochem. Cell Biol.

[6] Timasheff S.N. (1995). Solvent stabilization of protein structure. Meth. Mol. Biol.

[7] Bolen D.W., Baskakov I.V. (2001). The osmophobic effect: Natural selection of a thermodynamic force in protein folding. J. Mol. Biol.

[8] Singh L.R., Poddar N.K., Dar T.A., Kumar R., Ahmad F. (2011). Protein and DNA destabilization by osmolytes: The other side of the coin. Life Sci.

[9] Orsini G., Goldberg M.E. (1978). The renaturation of reduced chymotrypsinogen A in guanidine HCl. Refolding *versus* aggregation. J. Biol. Chem.

[10] Maeda Y., Koga H., Yamada H., Ueda T., Imoto T. (1995). Effective renaturation of reduced lysozyme by gentle removal of urea. Protein Eng.

[11] Yancey P.H., Somero G.N. (1979). Counteraction of urea destabilization of protein structure by methylamine osmoregulatory compounds of elasmobranch fishes. Biochem. J.

[12] Santoro M.M., Liu Y., Khan S.M.A., Hou L.X., Bolen D.W. (1992). Increased thermal stability of proteins in the presence of naturally occurring osmolytes. Biochemistry.

[13] Anjum F., Rishi V., Ahmad F. (2000). Compatibility of osmolytes with Gibbs energy of stabilization of proteins. Biochim. Biophys. Acta.

[14] Nayak A., Lee C.-C., McRae G.J., Belfort G. (2009). Osmolyte controlled fibrillation kinetics of insulin: New insight into fibrillation using the preferential exclusion principle. Biotechnol. Prog.

[15] Jamal S., Poddar N.K., Singh L.R., Dar T.A., Rishi V., Ahmad F. (2009). Relationship between functional activity and protein stability in the presence of all classes of stabilizing osmolytes. FEBS J.

[16] Kaushik J.K., Bhat R. (2003). Why Is Trehalose an Exceptional Protein Stabilizer?. J. Biol. Chem.

[17] Granata V., Palladino P., Tizzano B., Negro A., Berisio R., Zagari A. (2006). The effect of the osmolyte trimethylamine N-oxide on the stability of the prion protein at low pH. Biopolymers.

[18] Natalello A., Liu J., Ami D., Doglia S.M., de Marco A. (2009). The osmolyte betaine promotes protein misfolding and disruption of protein aggregates. Protein Struct. Funct. Bioinforma.

[19] Singh L.R., Dar T.A., Rahman S., Jamal S., Ahmad F. (2009). Glycine betaine may have opposite effects on protein stability at high and low pH values. Biochim. Biophys. Acta.

[20] Hincha D.K. (2006). High concentrations of the compatible solute glycinebetaine destabilize model membranes under stress conditions. Cryobiology.

[21] Poddar N.K., Ansari Z.A., Singh R.K.B., Moosavi-Movahedi A.A., Ahmad F. (2008). Effect of monomeric and oligomeric sugar osmolytes on Δ*G*d, the Gibbs energy of stabilization of the protein at different pH values: Is the sum effect of monosaccharide individually additive in a mixture?. Biophys. Chem.

[22] Scaramozzino F., Peterson D.W., Farmer P., Gerig J.T., Graves D.J., Lew J. (2006). TMAO promotes fibrillization and microtubule assembly activity in the C-terminal repeat region of tau. Biochemistry.

[23] Munishkina L.A., Henriques J., Uversky V.N., Fink A.L. (2004). Role of protein-water interactions and electrostatics in alpha-synuclein fibril formation. Biochemistry.

[24] Fung J., Darabie A.A., McLaurin J. (2005). Contribution of simple saccharides to the stabilization of amyloid structure. Biochem. Biophys. Res. Commun.

[25] Samuel D., Ganesh G., Yang P.-W., Chang M.-M., Wang S.-L., Hwang K.-C., Yu C., Jayaraman G., Kumar T.K.S., Trivedi V.D., Chang D.-K. (2000). Proline inhibits aggregation during protein refolding. Protein Sci.

[26] Ignatova Z., Gierasch L.M. (2006). Inhibition of protein aggregation *in vitro* and *in vivo* by a natural osmoprotectant. Proc. Natl. Acad. Sci. USA.

[27] Borwankar T., Röthlein C., Zhang G., Techen A., Dosche C., Ignatova Z. (2011). Natural osmolytes remodel the aggregation pathway of mutant huntingtin exon 1. Biochemistry.

[28] Uversky V.N., Li J., Fink A.L. (2001). Trimethylamine-*N*-oxide-induced folding of α-synuclein. FEBS Lett.

[29] Morar A.S., Olteanu A., Young G.B., Pielak G.J. (2001). Solvent-induced collapse of α-synuclein and acid-denatured cytochrome c. Protein Sci.

[30] Bomhoff G., Sloan K., McLain C., Gogol E.P., Fisher M.T. (2006). The effects of the flavonoid baicalein and osmolytes on the Mg 2+ accelerated aggregation/fibrillation of carboxymethylated bovine 1SS-α-lactalbumin. Arch. Biochem. Biophys.

[31] Nandi P.K., Bera A., Sizaret P.Y. (2006). Osmolyte trimethylamine N-oxide converts recombinant α-helical prion protein to its soluble β-structured form at high temperature. J. Mol. Biol.

[32] Tatzelt J., Prusiner S., Welch W. (1996). Chemical chaperones interfere with the formation of scrapie prion protein. EMBO J.

[33] Yang D.-S., Yip C.M., Huang T.H.J., Chakrabartty A., Fraser P.E. (1999). Manipulating the amyloid-β aggregation pathway with chemical chaperones. J. Biol. Chem.

[34] Scheibel T., Lindquist S.L. (2001). The role of conformational flexibility in prion propagation and maintenance for Sup35p. Nat. Struct. Mol. Biol.

[35] Kim H.Y., Kim Y., Han G., Kim D.J. (2010). Regulation of *in vitro* Aβ1–40 Aggregation mediated by small molecules. J. Alzheimer’s Dis.

[36] Liu R., Barkhordarian H., Emadi S., Park C.B., Sierks M.R. (2005). Trehalose differentially inhibits aggregation and neurotoxicity of beta-amyloid 40 and 42. Neurobiol. Dis.

[37] Qi W., Zhang A., Good T.A., Fernandez E.J. (2009). Two disaccharides and trimethylamine *N*-oxide affect Aβ aggregation differently, but all attenuate oligomer-induced membrane permeability. Biochemistry.

[38] Singer M.A., Lindquist S. (1998). Multiple effects of trehalose on protein folding *in vitro* and *in vivo*. Mol. Cell.

[39] Tanaka M., Machida Y., Niu S., Ikeda T., Jana N.R., Doi H., Kurosawa M., Nekooki M., Nukina N. (2004). Trehalose alleviates polyglutamine-mediated pathology in a mouse model of Huntington disease. Nat. Med.

[40] Kanapathipillai M., Lentzen G., Sierks M., Park C.B. (2005). Ectoine and hydroxyectoine inhibit aggregation and neurotoxicity of Alzheimer’s β-amyloid. FEBS Lett.

[41] Hudson S.A., Ecroyd H., Kee T.W., Carver J.A. (2009). The thioflavin T fluorescence assay for amyloid fibril detection can be biased by the presence of exogenous compounds. FEBS J.

[42] Liu K.-N., Wang H.-Y., Chen C.-Y., Wang S. (2010). l-Arginine reduces thioflavin T fluorescence but not fibrillation of bovine serum albumin. Amino Acids.

[43] Pedersen J.S., Dikov D., Flink J.L., Hjuler H.A., Christiansen G., Otzen D.E. (2006). The changing face of glucagon fibrillation: Structural polymorphism and conformational imprinting. J. Mol. Biol.

[44] Arora A., Ha C., Park C.B. (2004). Inhibition of insulin amyloid formation by small stress molecules. FEBS Lett.

[45] Ueda T., Nagata M., Imoto T. (2001). Aggregation and chemical reaction in hen lysozyme caused by heating at pH 6 are depressed by osmolytes, sucrose and trehalose. J. Biochem.

[46] Kim Y.-S., Cape S.P., Chi E., Raffen R., Wilkins-Stevens P., Stevens F.J., Manning M.C., Randolph T.W., Solomon A., Carpenter J.F. (2001). Counteracting effects of renal solutes on amyloid fibril formation by immunoglobulin light chains. J. Biol. Chem.

[47] Tanaka M., Machida Y., Nukina N. (2005). A novel therapeutic strategy for polyglutamine diseases by stabilizing aggregation-prone proteins with small molecules. J. Mol. Med.

[48] Dong X.-Y., Liu J.-H., Liu F.-F., Sun Y. (2009). Self-interaction of native and denatured lysozyme in the presence of osmolytes, l-arginine and guanidine hydrochloride. Biochem. Eng. J.

[49] Pedersen J.S., Andersen C.B., Otzen D.E. (2010). Amyloid structure—One but not the same: The many levels of fibrillar polymorphism. FEBS J.

[50] Hochacka P.W., Somero G.N. (2002). Water-Solute Adaptations: The Evolution and Regulation of the Internal Milieu.

[51] Pedersen J.S., Dikov D., Flink J.L., Hjuler H.A., Christiansen G., Otzen D.E. (2006). The changing face of glucagon fibrillation: Structural polymorphism and conformational imprinting. J. Mol. Biol.

[52] Ghodke S., Nielsen S.B., Christiansen G., Hjuler H.A., Flink J., Otzen D.E. (2011). Mapping out the multi-stage fibrillation of glucagon. FEBS J.

[53] Macchi F., Hoffmann S.V., Carlsen M., Vad B., Imparato A., Rischel C., Otzen D.E. (2011). Mechanical stress affects glucagon fibrillation kinetics and fibril structure. Langmuir.

[54] Macchi F., Hoffmann S.V., Carlsen M., Vad B., Imparato A., Rischel C., Otzen D.E. (2011). Mechanical Stress Affects Glucagon Fibrillation Kinetics and fibril Structure. Langmuir.

[55] Woody R.W. (1994). Contributions of tryptophan side chains to the far-ultraviolet circular dichroism of proteins. Eur. Biophys. J.

[56] Andersen C.B., Hicks M.R., Vetri V., Vandahl B., Rahbek-Nielsen H., Thøgersen H., Thøgersen I.B., Enghild J.J., Serpell L.C., Rischel C. (2010). Glucagon fibril polymorphism reflects differences in protofilament backbone structure. J. Mol. Biol.

[57] Pedersen J.S., Dikov D., Otzen D.E. (2006). *N*- and *C*-terminal hydrophobic patches are involved in fibrillation of glucagon. Biochemistry.

[58] Uversky V.N., Cooper M.E., Bower K.S., Li J., Fink A.L. (2002). Accelerated α-synuclein fibrillation in crowded milieu. FEBS Lett.

[59] Sasahara K., McPhie P., Minton A.P. (2003). Effect of dextran on protein stability and conformation attributed to macromolecular crowding. J. Mol. Biol.

